# Observations on the Use of Iodine as a Remedy for Bronchocele

**Published:** 1820-12

**Authors:** 


FOR THE LONDON MEDICAL AND PHYSICAL JOURNAL.

*Observations on the Use of Iodine as a Remedy for Bronchocele.* By Dr. Coindet. (Originally read to the Helvetian Physical Society, July 25, 1820.)

ABOUT a year since, when looking for a formula in the work of Cadet de Gassicourt, I found that Russel recommended the *varec (fucus vesiculosus)* as a remedy for bronchocele, under the name of *æthiops vegetalis*. Not knowing what relation there might exist between this plant and sponge, I suspected, from analogy, that iodine might be the active principle common to those two substances. I tried the iodine; and the astonishing success I obtained from it, encouraged me to pursue my enquiries.*

Hitherto burned sponge has formed the base of all the remedies which have had any success against bronchocele. It was Arnold of Villeneuve who made it known. It has been given in the form of wines, lozenges, powders, &c.; almost always combined with tonic medicines, to remedy its bad effects on the stomach: but, whatever correction be employed, it gives rise to spasms or cramps of that organ, which often remain for a long time after the use of the medicine has been discontinued, and which in some cases become a chronic disease very difficult of cure. These inconveniences happen especially when the bronchocele is voluminous, and as the patient is more remote from adult age; for the preparations of sponge but rarely produce these bad effects in children, where the bronchocele is but small, or of recent origin. These cramps had been attributed

---

* We omit a part of this memoir, containing observations on the appearances, size, &c. of bronchocele, and its probable causes, from its not comprising any thing novel of importance, excepting the following: "Two different causes have evidently appeared to me to be productive of bronchocele at Geneva: the first is the use of hard waters, or the pump-water of the low streets of this city; they produce bronchocele in a very short space of time. Thus the soldiers of the garrison, principally composed of young men, strangers to the canton, on drinking those waters, become affected with it in a manner as remarkable as it is sudden. This form of the malady, rarely severe, promptly disappears of itself on the drink being changed: distilled water prevents its increase, and even contributes to its diminution. I have remarked that this cause often leaves a *principle of bronchocele*, which is developed at some later period."

There is an apparent incongruity in the foregoing paragraph, (where the author says the bronchocele disappears of itself on the drink being changed, and then adds, that distilled water "prevents its increase" only, or that it "even contributes to its diminution;") but we have given a precise translation of the original.—Edit.

to the disappearance of the bronchocele; but they probably depend on some peculiar, unknown, combination, which may exist in the burned sponge, since iodine does not produce any such effects, and it causes bronchoceles to disappear more rapidly than burned sponge or any of its preparations, and it also removes more voluminous bronchoceles than those which can be cured by those medicines.

What is the substance in sponge that acts in a specific manner against bronchocele? It appears to me to be probable that it is iodine. I was confirmed in this opinion when I learned that Mr. Fife, of Edinburgh, had found iodine in sponge towards the end of the year 1819; when I had for six months witnessed its surprising effects in this disease.

Iodine exists in so small a proportion in sponge, that it is impossible to determine it. I have used that obtained from the mother waters of varec. It does not appear to form one of the constituent parts of marine productions: it would appear that it is only accidentally mingled with them, since it does not exist in the alkalies prepared in Sicily, Spain, and the Roman States. Sponge washed and macerated before it is submitted to analysis, presents a less quantity than when it has not been macerated. A property of this substance, yet so imperfectly known, is to form an acid when it combines either with oxygen or with hydrogen. The salts which result from its combination with oxygen being but little soluble in water, I have not used: I have preferred those obtained by its union with hydrogen, with which iodine has such an affinity, that it seizes it wherever it finds it, and forms with it an acid known by the name of *hydriodic acid.* It saturates all the bases and forms neutral salts, amongst which I have chosen as a medicine the *hydriodate of potash.* I have used that of soda with equal success. The hydriodate of potash is a deliquescent salt, forty-eight grains of which in an ounce of distilled water represent about thirty-six grains of iodine. It is this preparation, in this dose, which I have most frequently used.

The solution of this salt in a sufficient quantity of water may still dissolve iodine, and thus form an *iodurated hydriodate of potash;* a property which I have taken advantage of, for the purpose of increasing the strength of the remedy, in cases where a very hard, voluminous, and old, bronchocele appeared to resist the power of the simple saline solution; and by this means I have often obtained the most remarkable cures.

Iodine dissolves in a certain proportion of ether and in spirit of wine. Mr. Gay-Lussac found that water dissolved but the 1-7000th part of its weight.

An ounce of spirit of wine of 35° strength, dissolves, in a temperature of 15° of Reaumur, (65° Fahrenheit,) and under the ordinary degree of atmospheric pressure, sixty grains of iodine, or about a ninth of its weight; at 40° of concentration, and under the same conditions, it dissolves eighty-four grains, or about one-sixth: from which it appears that spirit of wine dissolves more or less according to the greater or less degree of its rectification.

In order to avoid all error in respect to the dose of the medicine in the following preparation, which I have termed *tincture of iodine*, I have employed forty-eight grains of iodine to one ounce of spirit of wine of 35°. I have used this preparation more than the preceding ones, (perhaps with superior success,) because it is easily prepared in the smallest towns, where there are not always chemists sufficiently experienced to obtain the saline hydriodates in a state of purity, and I have thought it right to make it the principal subject of my enquiries into the effects of a remedy which should come into general use.

This tincture should not be prepared at once in very large quantities, because it cannot be kept long without a deposition of chrystals of iodine taking place from it: besides, the large quantity of hydrogen contained in alcohol, and the extreme affinity of that substance for iodine, gives rise to a conversion of the tincture into iodurated hydriodic acid; a remedy, undoubtedly, very powerful: but, as there are in certain cases some reasons for the choice, in preference, of one of the three preparations I have mentioned, each of them should be such as the physician desires it, in order that he may direct the treatment with most surety, and obtain from it the most correct results.

I prescribe for adults ten drops of one of the three preparations, in half a tumbler of syrup of capillaire and water, to be taken in the morning fasting; a second dose at ten o'clock; and a third in the evening, or when the patient is going to bed.

Towards the end of the first week, I prescribe fifteen drops of it, instead of ten, three times a-day. Some days later, when the iodine has had a sensible effect on the tumor, I still increase the dose to twenty drops three times a-day, to sustain the action of it. Twenty drops contain about a grain of iodine. I have but rarely exceeded this dose: it has been sufficient to dissipate the most voluminous bronchoceles, when they were only an excessive development of the thyroid *body** without any other organic lesion.

After the treatment has been continued for eight days, the skin over the tumor becomes less tense, it is as it were thickened; the tumor becomes softer, in the first instance, before it diminishes in size; the tumors, when several are present, become more distinct, more separated from each other; they

* Dr. C. objects to its being termed a gland, because there is no evidence of its being such an organ.—Edit.

continue to soften and gradually waste away; in some instances, the nucleus of them, or, more correctly speaking, the part which has suffered organic lesion, becomes harder, it diminishes, and is isolated; and some of them become movable in proportion as the surrounding parts are removed by the influence of the iodine, which is an important circumstance, because in those severe cases where an operation for their removal is necessary, the diminished bulk of the tumor, and the consequent lessening of the size of the blood-vessels distributed to it, render the operation less difficult and less dangerous. Some cases of tumors, which were apparently bronchoceles, have resisted the action of this remedy, under whatever form, and for whatever period, I have administered it. I have reason to believe that these tumors were not proper bronchoceles, or that almost the whole of their extent had suffered some organic lesion.

In some cases, the cellular tissue which surrounds the tumor remains tumid, and conveys by the touch the sensation of an empty cyst.

The bronchocele is often incompletely removed, though it is lessened sufficiently to render it no longer incommodious nor a cause of deformity.

In a great number of cases, it subsides and is destroyed in the space of from six to ten weeks, so as to leave no trace of its existence.

In order that the precise effects of this remedy might be obtained, I have avoided the use of any local application, as bags, collars, &c.; means which, by the compression they exert, as well as by the saline or resolutive substances which enter into their composition, are not without some degree of efficacy.

Iodine is a stimulant; it gives tone to the stomach, excites the appetite: it neither influences the stools nor urine, nor does it provoke sweating, but it exerts its action expressly on the generative system, and especially on the uterus.* Administered in a certain dose, continued for some time, it is one of the most active emenagogues I know: it is, perhaps, by this sympathetic action that it cures bronchocele in a great proportion of cases.

I have employed it with complete success in one of those cases of chlorosis for which I should have prescribed myrrh, preparations of iron, &c. if I had not suspected this peculiar action. This substance merits, in this new point of view, the attention of physicians; and I have no doubt but that it will become, in able hands, one of the most powerful of the remedies with which modern chemistry has enriched the materia medica.

* Dr. C. thinks that bronchocele is especially connected with a certain state the genital organs, from the influence of which it originates.

